# Impact of Surgical Table Orientation on Flow Disruptions and Movement Patterns during Pediatric Outpatient Surgeries

**DOI:** 10.3390/ijerph18158114

**Published:** 2021-07-31

**Authors:** Anjali Joseph, David Neyens, Sahar Mihandoust, Kevin Taaffe, David Allison, Vishnunarayan Prabhu, Scott Reeves

**Affiliations:** 1 Center for Health Facilities Design and Testing, School of Architecture, College of Architecture, Arts and Humanities, Clemson University, Clemson, SC 29634, USA; smihand@clemson.edu (S.M.); adavid@clemson.edu (D.A.); 2 Department of Industrial Engineering, College of Engineering, Computing and Applied Sciences, Clemson University, Clemson, SC 29634, USA; dneyens@clemson.edu (D.N.); taaffe@clemson.edu (K.T.); vgirish@clemson.edu (V.P.); 3 Anesthesia and Perioperative Medicine, Medical University of South Carolina, Charleston, SC 29425, USA; reevess@musc.edu

**Keywords:** operating room, surgical table, flow disruptions, layout, movement, pediatric surgery

## Abstract

(1) Background: The surgical table within a typical ambulatory surgery operating room is frequently rotated and placed in different orientations to facilitate surgery or in response to surgeon preferences. However, different surgical table orientations can impact access to different work zones, areas and equipment in the OR, potentially impacting workflow of surgical team members and creating patient safety risks; (2) Methods: This quantitative observational study used a convenience sample of 38 video recordings of the intraoperative phase of pediatric outpatient surgeries to study the impacts of surgical table orientation on flow disruptions (FDs), number of contacts between team members and distance traveled; (3) Results: This study found that the orientation of the surgical table significantly influenced staff workflow and movement in the OR with an angled surgical table orientation being least disruptive to surgical work. The anesthesia provider, scrub nurse and circulating nurse experienced more FDs compared to the surgeon; (4) Conclusions: The orientation of the surgical table matters, and clinicians and architects must consider different design and operational strategies to support optimal table orientation in the OR.

## 1. Introduction

Optimal patient positioning is a critical part of any surgical procedure and the wrong position of the patient in the operating room (OR) during surgery can cause patient harm and injury with effects ranging from minor aches, pains and skin abrasions to paralysis and loss of life [1,2,3]. The patient is usually positioned during surgery to facilitate maximum exposure of the surgical site for the surgeon while at the same time providing access to patient airways, Intravenous (IV) lines and monitoring devices for the anesthesiologist. Several papers provide guidelines for different types of patient positions (e.g., supine, lateral decubitus, prone, etc.) and equipment needs (table or gurney type, attachments, pillows, mattress) to support the correct position and minimize complications and harm during surgery [2,3,4,5]. Some of these guidelines suggest that in addition to changing the configuration of the surgical table (e.g., height, incline, attachments) to accommodate a specific patient position, the surgical table may also need to be rotated to provide optimal access to the patient [6].

Operating rooms for general surgeries are usually designed around a standard position of the surgical table centered and parallel to the walls of the room with the assumption that most surgeries would be conducted in this orientation, with table rotation required for a smaller set of surgery types. In this standard position, the anesthesiologist is usually located at the head of the patient with the surgeon and scrub nurse located on either side of the patient depending on the surgical site location. The organization of fixed and difficult-to-move equipment and storage are usually optimized for this position of the surgical table. However, the surgical table is often rotated to accommodate different surgery types or surgeon preferences, requiring modifications to the position of the anesthesia workspace and moveable equipment associated with anesthesia and surgical activities as well as the surgical team members during surgery. These different orientations of the surgical table may pose workflow challenges and patient safety risks to the patient.

**Figure 1:**
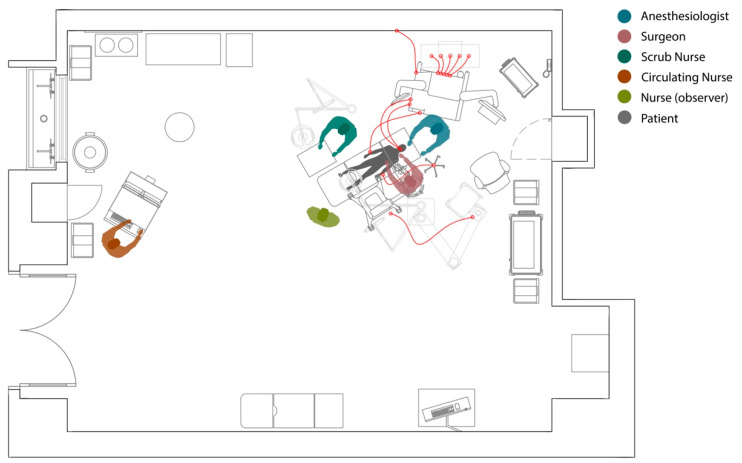
Multiple connections between the patient and OR equipment making surgical table rotations complicated and challenging.

In a highly interconnected and complex system like the operating room, a significant change or disruption to one part of the system potentially impacts other parts as well. For example, a particular orientation of the surgical table might result in more workspace or easier access to the surgical site for the surgeon while resulting in crowding for the scrub nurse or increasing the distance between anesthesia monitoring devices and the patient, creating challenges for the anesthesia team and safety risks for the patient. Figure 1 shows all the connections between the patient and different pieces of equipment in the OR during surgery. Though a few studies cite examples of patient harm related to surgical table rotation, there is a lack of research on the workflow challenges associated with different types of surgical table orientations [7,8]. The purpose of this study is to use a systems framework to understand how different types of surgical table positions may impact workflow challenges for OR team members.

The operating room environment is a highly complex system, and efficient and safe delivery of patient care depends on understanding and supporting the dynamic and changing relationships between the surgical team members, patient, tasks, equipment and the physical environment of the OR. Several studies show that disruptions are frequent during surgical procedures and poor room and equipment ergonomics are major contributing factors [9]. Specifically, small and cluttered ORs, large number of people in the OR, high traffic in and out of the OR, inadequate utilization of space and inefficient placement of equipment create risks for flow disruptions [9]. These flow disruptions (FDs) are defined as deviations from the natural progression of a procedure that potentially compromise safety or efficiency [10,11]. High rates of FDs in the OR have been associated with higher perceived workload for surgical team members [12], higher stress [13], increased surgery duration [14], increased errors [10,15] and increased patient mortality [16].

A recent study showed that the rate of major FDs in the OR increased as the rate of minor FDs increased, and this was particularly true for disruptions that involved OR equipment [17]. This study also found that more minor and major FDs took place in the anesthesia work area, compared to the other parts of the OR [17]. However, this study did not consider the impact of surgical table orientation on major and minor FDs. Flow disruptions are often indicative of underlying problems or latent conditions in the work system [17]. It is important to understand the nature of these FDs in the OR in order to identify and mitigate the inherent problems in the OR work system that impact staff workflow and patient safety. Given that surgical table rotation during surgery typically involves several pieces of equipment including the surgical table, instrument table and anesthesia equipment and significantly impacts the work of the surgical team, especially the scrub nurse, anesthesia providers and surgeon, there is a critical need to address the impact of different surgical table orientations on workflow disruptions and patient safety risks.

Certain orientations of the surgical table may result in some areas and access to certain equipment or workspaces in the OR being obstructed during the intraoperative phase, requiring team members to take alternative and undesirable paths to get to equipment or storage. This may lead to unnecessary travel within the OR and unwanted contacts between team members. For example, non-sterile team members (circulating nurse or anesthesia provider) may need to pass near sterile team members (surgeon and scrub nurse) to get to storage and equipment. Taaffe et al. [18] found that microbial load in the OR was higher in high traffic areas, highlighting the importance of laying out the OR to move high traffic flows away from the sterile zones around the surgical table. One study, using simulation modeling to test the impact of three different surgical table orientations on staff movement and contacts in the OR, found that an angled surgical table orientation with a mobile circulating nurse workstation at the foot of the table allowed for ease of movement for staff across the room without increasing unwanted contacts between team members [19]. This study also found that the number of contacts as well as distance traveled increased as the number of team members in the OR increased. However, this study did not examine the specific workflow disruptions, contacts and distance traveled by individual team members when conducting surgery in different table orientations.

This study utilizes the Systems Engineering Initiative for Patient Safety 2.0 (SEIPS 2.0) framework [20] to study the impacts of surgical table orientations on workflow and movement challenges experienced by surgical team members during outpatient pediatric procedures. The SEIPS 2.0 framework is based on a human-centered approach to improving patient safety that examines the dynamic interrelationships between tools and technology, people, tasks, organization and the built environment in a given healthcare system, the impact of this system on processes and how these processes in turn impact patient and staff safety outcomes [20]. Further, this dynamic work system constantly adapts in response to changing work systems factors, processes and outcomes. The primary questions addressed in this study are:

1. How do different types of surgical table orientations impact the workflow of surgical team members?
2. How do different types of surgical table orientations impact the overall movement of surgical team members during the intraoperative phase of the surgery?

## 2. Materials and Methods

This observational study used a convenience sample of 38 video recordings of pediatric outpatient surgeries from four different ORs at the Medical University of South Carolina (MUSC). This study complied with the American Psychological Association Code of Ethics and was approved by the Institutional Review Board at MUSC where observations were conducted (study ID: Pro00048787).

The video recordings of surgeries were captured using four video cameras located in four corners of the operating room such that all parts of the OR were visible. The video recordings were initiated when the patient entered the room and ended when the patient exited the room. The videos were then coded for surgery phases, surgical table orientation, team member locations, and flow disruptions using Noldus Observer XT V.12. Two researchers with human factors training coded the videos. All coders observed a set of 8 pre-recorded surgeries from a different OR and familiarized themselves with the coding scheme prior to coding. Coders also participated in two training sessions where they received education from human factors and clinical experts on human factors issues in the OR and also the overall goals and protocol related to the current study. The coding was conducted in parallel over three rounds until there was consensus. Percentage agreement over 83% was obtained for flow disruption and location codes using an index of concordance.

**Figure 2:**
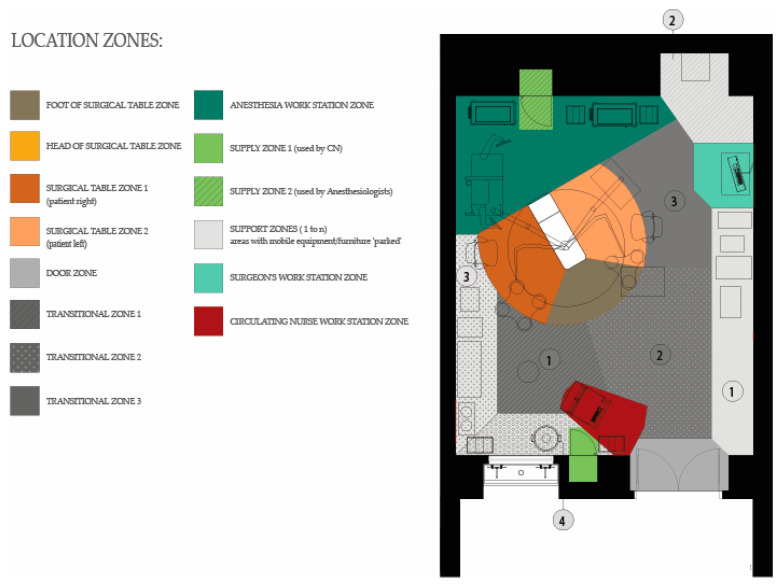
Location and type of zones in the ORs.

**Figure 3:**
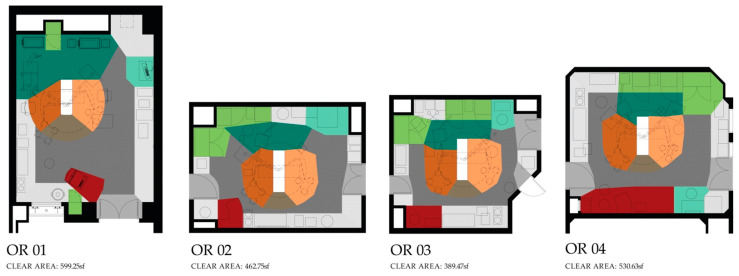
Floor plans of the four ORs observed with zones demarcated (refer to Figure 2 for legend). All floor plans are shown with surgical tables in orientation B.

Each video recording was first coded to mark different surgery phases (preoperative, intra-operative and post-operative) such that the duration of each phase could be obtained and the analysis could focus on activities within that phase. This study focuses on the intraoperative phase which was defined as the duration between the incision to the surgical site of the patient and the closure of the surgical site. The plan of the operating room was drawn to demarcate different functional zones, which were bounded and defined according to the type of function conducted in them [17]. The location of surgical team members at every point during the surgery was coded by marking the zone where they were located. Zones included the surgical table (head, foot, right of patient, left of patient), workstation, support, supply and door zones. Transition zones connect the functional zones and were primarily used for circulation within the OR (Figure 2). The head of the surgical table zone was only visible when the surgical table was rotated away from the anesthesia zone. All ORs observed as part of this study included all the functional and transitional zones. However, the ORs varied slightly in terms of sizes and the physical location of the zones within the OR and the size of zones (Figure 3).

### 2.1. Surgical Table Orientation

**Figure 4:**
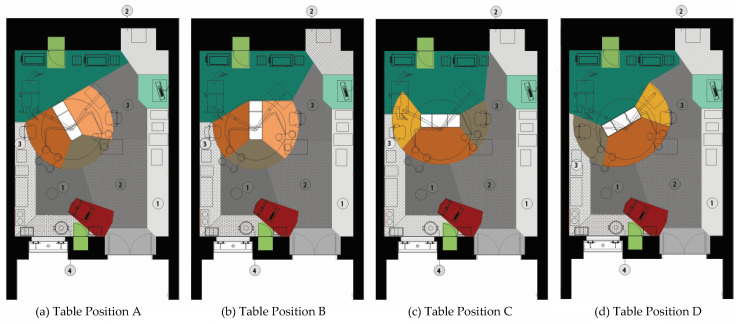
The four different surgical table orientations observed in the ORs in this study (**a**) Table position A, (**b**) Table position B, (**c**) Table position C, and (**d**) Table position D. These figures show the different surgical table orientations in OR 1. Refer to Figure 2 for legend.

Four different table orientations were identified: (A) the surgical table angled in the room with the head of the table in the anesthesia zone (orientation A), (B) parallel to the long or short walls (depending on shape of room) of the room with the head of the surgical table in the anesthesia zone (orientation B), (C) parallel to the long or short walls of the room (depending on room shape) with the anesthesia zone perpendicular to the head of the patient (orientation C) or (D) angled in the room with the anesthesia zone adjacent to the head of the surgical table (orientation D) (Figure 4). The zone boundaries and zone locations (especially head and foot of surgical table) changed based on table orientation and thus, the specific plans associated with each table orientation were used as reference by coders. That is, if the table orientation changed, coders would use the zone plans associated with that particular orientation to code location of surgical team members (Figure 4).

### 2.2. Flow Disruptions

In this study, the impact of the different surgical table orientations on staff workflow was understood by studying FDs. Flow disruptions were coded for all surgical team members (surgeon, anesthesia provider, circulating nurse and scrub nurse) using an existing taxonomy developed by Palmer and colleagues [11] and adapted for this study. Five types of FDs were coded including layout, environmental hazards, usability, interruption and equipment failure. The definition of each type of FD can be found in Table 1. All FDs were further classified for severity of FD based on a taxonomy developed by Parker et al. [21]. Flow disruptions were classified into one of six categories: 1—no impact/minor disruption-no response; 2—momentary disruption (acknowledgement of disruption, no pause in task); 3—momentary distraction (short pause < 10 s); 4—primary task interrupted (task cessation > 10 s); 5—primary task disruption (secondary task engaged); 6-repeat task. Further, any FD that resulted in a pause or break in the primary activity being performed was classified as a ‘major’ FD (categories 3–6) and the rest were termed as ‘minor’ FDs (categories 1 and 2).

### 2.3. Surgical Team Movement

This study also focused on understanding how different surgical table orientations impact the movement of surgical team members during the intraoperative phase. The data obtained from video coding of the surgeries were used to create a playback simulation using AnyLogic simulation software. Data on staff movement and interaction during the intraoperative phase was obtained from the software platform which was customized to track the location of all team members with the required degree of precision and to obtain measures related to the overall movement of all team members such as the total distance traveled (m) and the total number of contacts per surgery. A contact was calculated by monitoring the distance between any two subjects in the operating room in the playback model. When another subject passed or was within a prespecified threshold of (0.6 m) of another, a contact was recorded.

### 2.4. Analyses

The event-based data around FDs obtained from the Noldus Observer XT 12 software (Noldus Information Technology, Wageningen, the Netherlands) were converted into time-based data with 1 s intervals to facilitate statistical analysis. Data on distance traveled and number of contacts was obtained from the AnyLogic simulation software program. Descriptive statistics were used to report characteristics of FDs and movement patterns (distance traveled and number of contacts) associated with different types of surgical table orientations across the intraoperative phases of the observed pediatric surgeries.

The analysis was conducted at the surgical team member level with data counting the number of FDs, the distance traveled, and the number of contacts for each person in the operating room during the intraoperative phase. Each person in the room was coded according to their role in the surgical team (e.g., scrub nurse, circulating nurse anesthesia provider, surgeon) and there could be more than one provider for each type during the surgical case.

Quasi-Poisson regression models were used to examine the impact that surgical table orientation had on the number of FDs. Quasi-Poisson regression is a generalization of the Poisson regression model and estimates the over-dispersion and does not assume that the mean and variance are equal. These models were used to predict the count of all FDs, the count of major FDs, and the count of minor FDs during the intraoperative phase of the surgery.

In addition to the overall FD analysis, binary indicator variables were created to identify if during the intraoperative phase each person in the OR experienced any flow disruption of a specific type. Specifically, the indicator variables were created to identify if a healthcare worker was involved in a layout FD, an environmental hazard FD, an interruption FD, an equipment FD, or a usability FD. Binary logistic regression models were used to evaluate the likelihood of an individual being involved in any of the specific FD types. Explanatory variables considered in the analysis included binary indicator variables for the surgical table orientation, the provider types, and the specific operating room where the surgery was conducted. The position of the surgical site was also accounted for since this might impact the position of the surgical team around the patient. The surgeries were categorized as upper body (e.g., head and neck) and lower body (e.g., chest, abdomen, pelvic). Additionally, the number of people in the operating room and the duration of the intraoperative phase were included as explanatory variables. All statistical analysis was conducted in R Studio version 1.4.1103 (R Studio, Boston, MA, USA). Stepwise deletion was used to remove insignificant parameters from the models.

## 3. Results

Thirty-eight pediatric surgeries conducted in four different ORs were video recorded and analyzed. Table 2 shows the distribution of the surgical cases across the four surgical table orientations (A, B, C and D) by OR characteristics, length of the intraoperative phase, position of surgical site as well as the number of team members involved in the surgery. The total observation time (intraoperative phase) across all surgeries was 823 min (average surgery length of 21.7 min ± 24.4). The average length of the intraoperative phase varied across the surgeries with those conducted in orientation A being on average longer (28.9 ± 22.8) than the those in the other orientations. Two-thirds of the cases observed involved upper body (head and neck) surgeries while the rest were lower body surgeries. Upper body surgeries observed included adenoids/tonsillectomy, ear tube surgery, laryngoscopy, bronchoscopy and esophageal dilation, Lower body surgeries included circumcision, catheter removal, hernia repair and gastrocutaneous fistula closure. Majority of the surgeries were conducted in OR 1 and most surgeries had around 5 surgical team members present.

### 3.1. Surgical Table Orientation and Flow Disruptions

Table 3 provides the distribution of flow disruptions across the four surgical table orientations during the intraoperative phase based on FD severity, FD type and the surgical team member involved. A total of 1001 flow disruptions were observed during the intraoperative phase across the 38 pediatric surgeries; an average of 1.77 ± 1.44 FD/min were observed across the surgeries. The average number of FDs/min ranged between 1.14 ± 1.07 (orientation A) to 2.15 ± 1.47 (orientation D). Around 27% of all FDs were major disruptions, with more major FDs/min observed in orientations B (0.59 ± 0.37) and D (0.67 ± 0.63) compared to orientation A (0.38 ± 0.52). A similar pattern was observed with minor disruptions. Layout related FDs were the most commonly observed type of FD (56.8%), followed by environmental hazards (26.5%) and interruptions (12.6%). Very few instances of equipment and usability related FDs were observed. The anesthesia provider (30.1%) and circulating nurse (27.9%) were each involved in little less than a third of the disruptions, followed by the scrub nurse (20.3%). The surgeon was involved in fewer disruptions compared to other team members (14.9%). However, the surgeon and scrub nurse (SN) were involved in more FDs/min in orientation D (surgeon = 1.02 ± 0.49, SN = 0.45 ± 0.40), compared to orientation A (surgeon = 0.25 ± 0.47, SN = 0.15 ± 0.21).

#### 3.1.1. Impact of Surgical Table Orientation on FDs

The quasi-Poisson regression model predicting all flow disruptions during the intraoperative surgical phase is shown in Table 4. The surgical table orientation within the OR significantly influenced the overall incidence rates of flow disruptions. Flow disruptions were 1.48 (95% CI: 1.09, 2.01) times higher when the surgical case used table orientation B and 1.79 (95%CI: 1.30, 2.47) times higher when the surgical case used table orientation D than for the other surgical table orientations controlling for all other variables. Anesthesia providers (RR = 1.97, 95% CI: 1.36, 2.89), circulating nurses (RR = 1.54 95% CI: 1.06, 2.28), and scrub nurses (RR = 1.52, 95% CI: 1.01, 2.30) experienced higher numbers of flow disruptions than surgeons.

#### 3.1.2. Impact of Surgical Table Orientation on Minor FDs

The quasi-Poisson regression model predicting minor flow disruptions during the intraoperative surgical phase is shown in Table 5. The surgical table orientation within the OR significantly influenced the incidence rates of minor flow disruptions. Minor flow disruptions were 1.42 (95% CI: 1.04, 1.94) times more frequent when the surgical case used table orientation B and 1.78 (95%CI: 1.29, 2.46) times more frequent when the surgical case used table orientation D than for the other surgical table orientations controlling for all other variables. Anesthesia providers experienced 1.62 (95% CI: 1.23, 2.12) times more minor FDs than the other individuals in the operating room. Surgeons experienced fewer minor FDs (RR = 0.59, 95% CI: 0.39, 0.85) than all other individuals in the operating room.

#### 3.1.3. Impact of Surgical Table Orientation on Major FDs

The quasi-Poisson regression model predicting major FDs during the intraoperative surgical phase is shown in Table 6. The surgical table orientation within the OR did not significantly impact the rate of major FDs. Major FDs were 1.78 (95% CI: 1.14, 2.71) times higher for scrub nurses than other individuals. When the surgical procedure was below the head or neck region of the patient the rate of major FDs were significantly less frequent (RR = 0.40 95% CI: 0.22, 0.69).

#### 3.1.4. Impact of Surgical Table Orientation on Type of FDs

The results of several binary logistic regression models suggest that individuals working in ORs with table orientation A were less likely to experience a layout FD (OR = 0.17, 95%CI: 0.07, 0.38), controlling for the duration of the surgery. Individuals working in surgeries with table orientation D were less likely to experience a usability FD (OR = 0.18, 95% CI: 0.03, 0.65) with circulating nurses also less likely to experience a usability FD (OR = 0.25, 95% CI: 0.06, 0.77). There were no significant table orientations that predicted the likelihood of individuals experiencing an equipment, environmental hazard, or an interruption FD during the intraoperative phase of the surgery.

### 3.2. Surgical Table Orientation and Distance Traveled in the OR

The total distance traveled by surgical team members during the intraoperative phase across all observed surgeries was 6776 m. Team members traveled an average of 178.3 m/surgery and 8.13 ± 3.9 m/min during the intraoperative phase of a surgery (Table 7). The circulating nurse walked the most during the intraoperative phase (4.5 m/min + 3.24), followed by the scrub nurse (1.8 m/min ± 2.24). The surgeon and anesthesia provider walked less than a meter/min during the intraoperative phase. The average distance/min traveled by team members was highest for orientation C followed by orientation A, then D and B. While a similar pattern was observed for the surgeon’s movement, no consistent pattern was observed for the other team members across the different surgical table orientations.

A logistic regression model predicting the likelihood that an individual was in the 4th quartile of estimated distance moved during the intraoperative surgical phase is shown in Table 8. The 4th quartile was used as an indication of identifying those individuals who moved more than 75% of the other individuals. If the surgical table was in orientation B, the individuals in the operating room were 2.83 (95% CI: 1.07, 7.83) times more likely to be in the 4th quartile of distance moved. That is, when the surgical table was in orientation B, the individuals were more likely to walk more than on the other orientations. During the intraoperative phase, circulating nurses and scrub nurses were more likely to be in the 4th quartile than the other provider types, which suggest that they walked much more than other providers in the operating room.

### 3.3. Surgical Table Orientation and the Number of Contacts

The total number of contacts recorded between team members during the intraoperative phase when they passed each other within a prespecified threshold (0.6 m) was 1789. Table 9 shows the number of contacts between surgical team members during the intraoperative phase across the four different surgical table orientations. The highest average number of contacts/min was recorded in orientation C (2.90 ± 2.21) and the least in orientation A (2.09 ± 1.53). The fewest number of contacts/min were recorded for the anesthesia provider, scrub nurse, and surgeon in orientation A, and for the circulating nurse in orientation D. The circulating nurse experienced the maximum number of contacts/min in orientation A.

The Quasi-Poisson regression model predicting the number of contacts for each individual in the OR during the intraoperative phase is shown in Table 10. Individuals experienced 2.82 (95% CI: 1.62, 4.81) times more contacts when working with the surgical table in orientation C than the other surgical table orientations. Operating room 4 was associated with significantly less contacts than the other operating rooms (RR = 0.36, 95% CI: 0.19, 0.35) and anesthesia providers experienced significantly less contacts than other providers (RR = 0.36, 95% CI: 0.23, 0.54). When there were more people in the operating room, there were significantly more contacts (RR = 1.19, 95% CI: 1.07, 1.31). Additionally, when the surgical site was below the head or neck (lower part of the body) there were 3.59 (95% CI: 2.57, 5.04) more contacts than if the surgery site was on the patient’s head or neck.

## 4. Discussion

This is the first quantitative observational study to examine how the work of the surgical team is impacted by different types of surgical table orientations. This study found that the orientation of the surgical table within the operating room significantly influenced staff workflow and movement in the OR during the intraoperative phase of surgery. More specifically, FDs during the intraoperative phase were 1.48 times higher when the surgical case used orientation B (surgical table in conventional orientation parallel to long or short walls with head of table in anesthesia zone) and 1.79 times higher with orientation D (angled in the room with the anesthesia zone adjacent to the head of the surgical table) after controlling for other variables. The findings were similar for minor FDs, though there was no impact of surgical table orientation on the number of major FDs. This study also demonstrated that surgical team members were less likely to experience a layout related FD in surgeries conducted in the angled surgical table orientation A (surgical table angled in room with head of the table in anesthesia zone) compared to all other orientations. Team members working in operating rooms with surgical table orientation C (parallel to the long or short walls of the room (depending on room shape) with the anesthesia zone perpendicular to the head of the patient) were 2.82 times more likely to experience contacts with other team members. The average number of contacts/min was least for all team members in orientation A. The study also found that individuals working in orientation B were more likely to walk longer distances during the intraoperative phase.

A key insight from this study is that surgical table orientation matters with the angled orientation A impacting fewer flow disruptions, specifically the occurrence of layout related disruptions. A previous simulation-based study found that the angled surgical table orientation was preferred as it provided space for movement in the room without increasing the number of contacts [19]. This observational study confirms the findings from this previous study and provides additional rationale for an angled surgical table orientation A by showing a reduction in flow disruptions, especially those related to layout. Further, orientation A did not significantly impact the movement patterns in the OR during the intraoperative phase compared to other orientations such as B and C. Using quantitative observational data from 38 pediatric surgeries, this study confirms that the angled surgical table orientation is less disruptive to surgical work while allowing optimal access to the patient, storage and equipment during surgery.

On the other hand, orientation B and D resulted in a higher number of FDs during the intraoperative phase. While table orientation B is close in configuration to the angled orientation A, there were significantly more FDs in this orientation. In ORs with narrow space available at the foot of the table (e.g., OR 2 and OR 3), the circulation space at the foot of the table may get blocked with equipment during the intraoperative phase requiring team members to find alternative (and often undesirable) paths to get to the storage and equipment. This may result in unwanted contacts, more disruptions and greater travel within the OR. The angling of the surgical table opens up space at the foot of the table, potentially helping to reduce some of these challenges. The higher number of FDs in orientation D can be explained by the spatial constraints of accommodating the surgeon and anesthesia provider and other stored equipment in a limited workspace in the corner of the room.

This study also found that surgical team members are impacted differently by different orientations of the surgical table. The surgeon and scrub nurse experienced fewer FD/min in orientation A compared to orientation D. Overall, the anesthesia provider, circulating nurse and scrub nurses experienced more FDs during the intraoperative phase compared to the surgeon. An interesting finding from this study is that the scrub nurse experienced 1.78 times more major FDs compared to other team members. This may suggest that while the position of the table is optimized for the surgeon, the scrub nurse who is assisting the surgeon from the opposite side of the table, may be constrained for space and may experience major disruptions to their work. This study also found that circulating nurses and scrub nurses walked much more than other providers in the OR. The movement of the circulating nurse in the OR is expected, given the requirements of their role to monitor and support the team during surgery. However, the scrub nurse is usually fairly stationary during the intraoperative phase and the movements of this individual (while less than that of the circulating nurse) may be indicative of layout challenges requiring frequent adjustments to accommodate equipment or other staff in the OR. This study also confirms findings from other OR studies that showed the negative impacts of increased number of people in the OR [19,22]. In this study, the number of contacts significantly increased when the number of people in the OR increased.

The findings from this study have significant implications for both OR design and clinical practice. This study suggests that OR designers and administrators should not only reconsider standard surgical table positions and associated locations of different functional zones in the design and layout of ORs, but should also evaluate layout and workflow in the context of different surgical table orientations. Simulation-based evaluations that allow teams to enact surgical procedures in different surgical table orientations in a physical mock-up may help in proactively identifying and mitigating workflow challenges [23]. In this study, an angled surgical table orientation was shown to be most supportive for all surgical team members during the intraoperative phase of general surgeries. However, other orientations may be feasible provided there is adequate space around the surgical table, especially at the foot of the surgical table, to support positioning of staff and equipment without obstructing movement in the OR.

From a clinical standpoint, this study highlights the workflow challenges experienced by surgical team members in different orientations. Some of these surgical table orientations may be required for certain types of surgeries. However, to the extent possible, surgeries should be conducted in standard surgical table orientations which support workflows for all team members. OR managers, administrators and clinicians should also be cognizant of spatial constraints posed in certain orientations such as C and D and prevent accumulation of equipment and clutter in the OR that may further exacerbate workflow challenges in these orientations. While this study did not measure incidence of patient harm or injury associated with different surgical table orientations, other studies have shown that high rates of FDs contribute to surgical errors [10,15] and increased patient mortality [16]. The surgical table orientation not only impacts staff workflow, but also has implications for patient safety.

This study is arguably the most detailed analysis of the relationship between surgical table orientation and surgical workflow in the OR ever conducted. By using a systems approach, we were able to study the dynamic interactions between surgical team members and their work in the context of different spatial layout conditions (table orientation). The type of data obtained from the 38 general pediatric surgeries is very extensive. However, this study has some limitations. While this study included data from all individual team members across these surgeries, it is a relatively small sample. Further, different types of pediatric surgeries were included in this sample. While the surgeries were categorized based on surgical site location given its relevance for positioning of team members, it is possible that specific variations among procedures could potentially confound findings. Given that this is the first extensive study of its kind on surgical table positioning and orientation in surgery and that table rotations are common in all types of ORs, there is a critical need to expand this work to other types of surgical environments.

## 5. Conclusions

The layout of the operating room, especially the position of the surgical table, significantly impacts the work of all surgical team members by impacting flow disruptions as well as movement in the operating room. Utilizing a systems approach, this study found that an angled surgical table orientation is optimal for supporting the work of all team members in general pediatric surgeries while other orientations may cause challenges to workflow and movement. There are key areas of improvement identified in this study that are relevant for architects as well as clinicians and administrators.

## Figures and Tables

**Table 1:** Definitions of different types of flow disruptions (FD).

Flow Disruption Types	Definition
Environmental Hazards	Incidents involving the interaction of surgical staff with the environment such as: Staff Slipping/falling/tripping Staff interaction with sharp objects and contaminated needles Collision between staff and objects Excessive reach for accessing patient, objects, or equipment
Layout	Spatial organization or positioning of certain items in the operating room that hinder the surgical staff member’s performance by blocking their route or impeding visibility. These items include: Connectors or wires Equipment or furniture Permanent structures or fixed equipment
Interruptions	Incidents unrelated to surgical procedures that distract the surgical staff: Incoming or outgoing calls from phones or pagers People who are external to the core surgical team Dropping or picking up items from the floor while conducting during an activity. Shift changes during surgical procedures Searching for missing items/supply/instrument during an activity Door openings during surgeries Interacting with personal phones
Equipment Failure	Incidents related to malfunctioning or broken equipment during surgical procedures
Usability	Problems associated with: Computers (operating software, programs, and utilities) Pointing devices Monitors Sterile field barriers (e.g., surgical drapes, gowns, or gloves), packaging materials (unwrapping or opening packaging containing supplies)

**Table 2:** Overview of surgeries observed across different surgical table orientations.

	Surgical Table Orientation				Total
	A	B	C	D	
Number of surgeries observed	9	13	4	12	38
Total length of intraoperative phase across all surgeries (min)	260	278	62	223	823
Average length of intraoperative phase (min), M ± SD	28.9 ± 22.8	21.4 ± 34.0	15.45 ± 7.8	18.5 ± 16.8	21.65 ± 24.41
Surgery site position					
Upper body (head/neck)	2	9	4	9	24
Lower body	7	4		3	14
Operating room number (area)					
OR 1 (599 sf)	9	8	4	3	24
OR 2 (463 sf)		4			4
OR 3 (389 sf)		1		8	9
OR 4 (531 sf)				1	1
Average number of staff members present during surgery, M ± SD	5.4 ± 2.4	5 ± 0.8	5.75 ± 2.1	4.6 ± 1.1	5.07 ± 1.5

**Table 3:** Flow disruptions across different surgical table orientations.

FD Characteristics	Surgical Table Orientation								Total	
	A		B		C		D			
	(F)	M ± SD	(F)	M ± SD	(F)	M ± SD	(F)	M ± SD	(F)	M ± SD
Number of FDs	21	1.14 ± 1.07	378	1.96 ± 1.19	89	1.54 ± 0.56	324	2.15 ± 1.47	1001	1.77 ± 1.24
**Flow Disruptions—By Severity**										
Major FDs	54	0.38 ± 0.52	104	0.59 ± 0.37	25	0.39 ± 0.17	86	0.67 ± 0.63	269	0.54 ± 0.49
Minor FDs	156	0.76 ± 0.58	274	1.37 ± 0.91	64	1.15 ± 0.43	238	1.47 ± 0.93	732	1.22 ± 0.83
**Flow Disruptions—By Type**										
Environmental Hazard FDs	64	0.33 ± 0.23	98	0.49 ± 0.35	16	0.27 ± 0.18	87	0.49 ± 0.33	265	0.42 ± 0.31
Layout-related FDs	96	0.56 ± 0.70	230	1.20 ± 0.88	61	1.06 ± 0.45	181	1.24 ± 0.91	568	1.04 ± 0.83
Interruption-related FDs	30	0.09 ± 0.09	36	0.15 ± 0.26	6	0.17 ± 0.09	54	0.41 ± 0.41	126	0.21 ± 0.30
Equipment failure FDs	5	0.10 ± 0.23	3	0.04 ± 0.11	0	0.01 ± 0.00	0	0.00 ± 0.00	8	0.04 ± 0.13
Usability FDs	14	0.06 ± 0.06	11	0.08 ± 0.12	6	0.06 ± 0.09	2	0.01 ± 0.03	33	0.05 ± 0.09
**Surgical Team Member Involvement in FDs**										
Surgeon	23	0.25 ± 0.47	44	0.42 ± 0.40	11	0.14 ± 0.28	71	0.67 ± 1.02	149	0.43 ± 0.67
AN provider	68	0.27 ± 0.18	137	0.51 ± 0.49	18	0.29 ± 0.12	78	0.49 ± 0.44	301	0.43 ± 0.39
Circulating nurse	83	0.32 ± 0.25	103	0.48 ± 0.36	39	0.61 ± 0.14	54	0.29 ± 0.50	279	0.39 ± 0.38
Scrub Nurse	36	0.15 ± 0.21	70	0.39 ± 0.56	19	0.25 ± 0.21	78	0.45 ± 0.40	203	0.34 ± 0.42

**Table 4:** Quasi-Poisson regression model predicting the number of major flow disruptions during the intraoperative phase.

Coefficient	Estimate	Std. Error	t Value	*p*-Value	Rate Ratios	95%CI on RR
Intercept	0.44	0.20	2.25	0.03		
Table Orientation B	0.39	0.16	2.52	0.01	1.48	(1.09, 2.01)
Table Orientation D	0.58	0.16	3.55	<0.001	1.79	(1.30, 2.47)
Anesthesia Provider	0.68	0.19	3.53	<0.001	1.97	(1.36, 2.89)
Circulating Nurse	0.43	0.19	2.22	0.03	1.54	(1.06, 2.28)
Scrub Nurse	0.42	0.21	1.99	0.05	1.52	(1.01, 2.30)
Duration	0.00	0.00	10.18	<0.001	1.00	(1.00, 1.00)
Dispersion	3.648					
Deviance at convergence	531.26	df = 107				
Deviance at intercept	905.23	df = 164				

**Table 5:** Quasi-Poisson regression model predicting the number of minor flow disruptions during the intraoperative phase.

Coefficient	Estimate	Std. Error	t-Statistic	*p*-Value	Rate Ratio	95%CI on RR
Intercept	0.49	0.15	3.342	0.001	1.63	(1.22, 2.16)
Table Orientation B	0.35	0.16	2.202	0.03	1.42	(1.04, 1.94)
Table Orientation D	0.58	0.16	3.507	<0.001	1.78	(1.29, 2.46)
Anesthesia	0.48	0.14	3.438	<0.001	1.62	(1.23, 2.12)
Surgeon	−0.53	0.20	−2.703	0.007	0.59	(0.39, 0.85)
Duration	0.00	0.00	10.281	<0.001	1.00	(1.00, 1.00)
Dispersion	2.772					
Deviance at convergence	426.01	df = 165				
Deviance at intercept	748.49	df = 170				

**Table 6:** Quasi-Poisson regression model predicting the number of major flow disruptions during the intraoperative phase.

Coefficient	Estimate	Std. Error	t-Statistic	*p*-Value	Rate Ratios	95%CI on RR
Intercept	−0.12	0.15	−0.795	0.43	0.88	(0.65, 1.19)
Scrub Nurse	0.58	0.22	2.627	0.009	1.78	(1.14, 2.71)
Surgical site on lower part of the body	−0.92	0.29	−3.217	0.002	0.40	(0.22, 0.69)
Duration	0.00	0.00	6.76	<0.001	1.00	(1.00, 1.00)
Dispersion	2.492					
Deviance at convergence	353.08	df = 167				
Deviance at intercept	466.35	df = 170				

**Table 7:** Distance traveled by surgical team members during the intraoperative phase across different surgical table orientations.

Surgical Team Member	Surgical Table Orientation								Total	
	A		B		C		D			
	D (m)	(D/min), M ± SD	D (m)	(D/min), M ± SD	D (m)	(D/min), M ± SD	D (m)	(D/min), M ± SD	D (m)	(D/min), M ± SD
All team members	2426.5	8.85 ± 4.18	1909.3	6.47 ± 3.61	752.4	11.47 ± 3.79	1688.1	8.28 ± 3.54	6776.4	8.13 ± 3.90
Surgeon	318.2	0.96 ± 0.96	170.08	0.87 ± 0.63	92.8	1.19 ± 2.37	205.4	0.87 ± 1.15	786.4	0.92 ± 1.09
Anesthesia provider	123.8	0.57 ± 0.48	238.59	1.11 ± 1.36	95.6	1.76 ± 0.70	130.2	0.57 ± 0.65	588.2	0.88 ± 0.98
Scrub nurse	296	1.13 ± 1.00	217.9	0.68 ± 0.86	141.7	1.83 ± 1.53	652.7	3.57 ± 3.07	1308.3	1.82 ± 2.24
Circulating nurse	1688.5	6.20 ± 2.98	1282.8	3.82 ± 2.44	422.3	6.69 ± 1.32	699.9	3.28 ± 3.96	4093.5	4.51 ± 3.24

**Table 8:** Logistic regression predicting the likelihood that an individual was in the fourth quartile of distanced moved during the intraoperative phase.

Coefficient	Estimate	Std. Error	z-Score	*p*-Value	Rate Ratios	95%CI on RR
Intercept	−4.61	0.74	−6.202	<0.001	0.01	(0.002, 0.04)
Table Orientation B	1.04	0.50	2.063	0.04	2.83	(1.07, 7.83)
Circulating Nurse	3.22	0.65	4.96	<0.001	25.07	(7.81, 103.63)
Scrub Nurse	2.57	0.68	3.753	<0.001	13.06	(3.68, 56.48)
Duration	0.00	0.00	4.422	<0.001	1.00	(1.00, 1.001)
Deviance at convergence	129.01	df = 166				
Deviance at intercept	192.87	df = 170				

**Table 9:** Number of contacts between surgical team members during the intraoperative phase across different surgical table orientations.

Surgical Table Orientation										
Surgical Team Member	A		B		C		D		Total	
	(F)	M ± SD	(F)	M ± SD	(F)	M ± SD	(F)	M ± SD	(F)	M ± SD
All team members	496	2.09 ± 1.53	572	2.15 ± 1.86	194	2.90 ± 2.21	527	2.58 ± 1.40	1789	2.35 ± 1.64
Surgeon	121	0.45 ± 0.47	100	0.54 ± 0.51	62	0.79 ± 1.58	152	0.89 ± 0.92	435	0.66 ± 0.79
Anesthesia provider	36	0.21 ± 0.21	74	0.42 ± 0.60	26	0.49 ± 0.25	57	0.30 ± 0.40	193	0.34 ± 0.43
Scrub nurse	77	0.35 ± 0.35	127	0.46 ± 0.77	55	0.70 ± 0.68	175	0.75 ± 0.62	434	0.55 ± 0.63
Circulating nurse	262	1.08 ± 0.85	271	0.72 ± 0.61	51	0.92 ± 0.39	143	0.65 ± 0.83	727	0.80 ± 0.72

**Table 10:** Quasi-Poisson regression model predicting the number of contacts for each individual in the OR during the intraoperative phase.

Coefficient	Estimate	Std. Error	t-Statistic	*p*-Value	Rate Ratios	95%CI on RR
Intercept	1.62	0.29	5.652	<0.001		
Table Orientation C	1.04	0.28	3.752	<0.001	2.82	(1.62, 4.81)
OR 4	−1.36	0.16	−8.467	<0.001	0.26	(0.19, 0.35)
Anesthesia	−1.02	0.22	−4.726	<0.001	0.36	(0.23, 0.54)
Number of people in OR	0.17	0.05	3.239	0.001	1.19	(1.07, 1.31)
Surgical site on lower part of the body	1.28	0.17	7.444	<0.001	3.59	(2.57, 5.04)
Dispersion	8.011					
Deviance at convergence	1320.0	df = 165				
Deviance at intercept	2527.7	df = 170				

## Data Availability

The data utilized in this study is part of the private data repository related to the patient safety learning lab, ‘Realizing Improved Patient Care through Human-centered Design in the OR (RIPCHD.OR).
